# Review of “*Mosquitoes of the World*” by Richard C. Wilkerson, Yvonne-Marie Linton, and Daniel Strickman

**DOI:** 10.1186/s13071-021-04848-6

**Published:** 2021-06-26

**Authors:** Bart G. J. Knols

**Affiliations:** K&S Consulting, Kalkestraat 20, 6669 CP Dodewaard, The Netherlands

## Book details

Wilkerson RC, Linton Y-M, Strickman D

*Mosquitoes of the World*

Johns Hopkins University Press, Baltimore; 2021, Vol. 1 and Vol. 2, 1332 pages

ISBN 978-1-421438-14-6

## Review

**Fig. 1:**
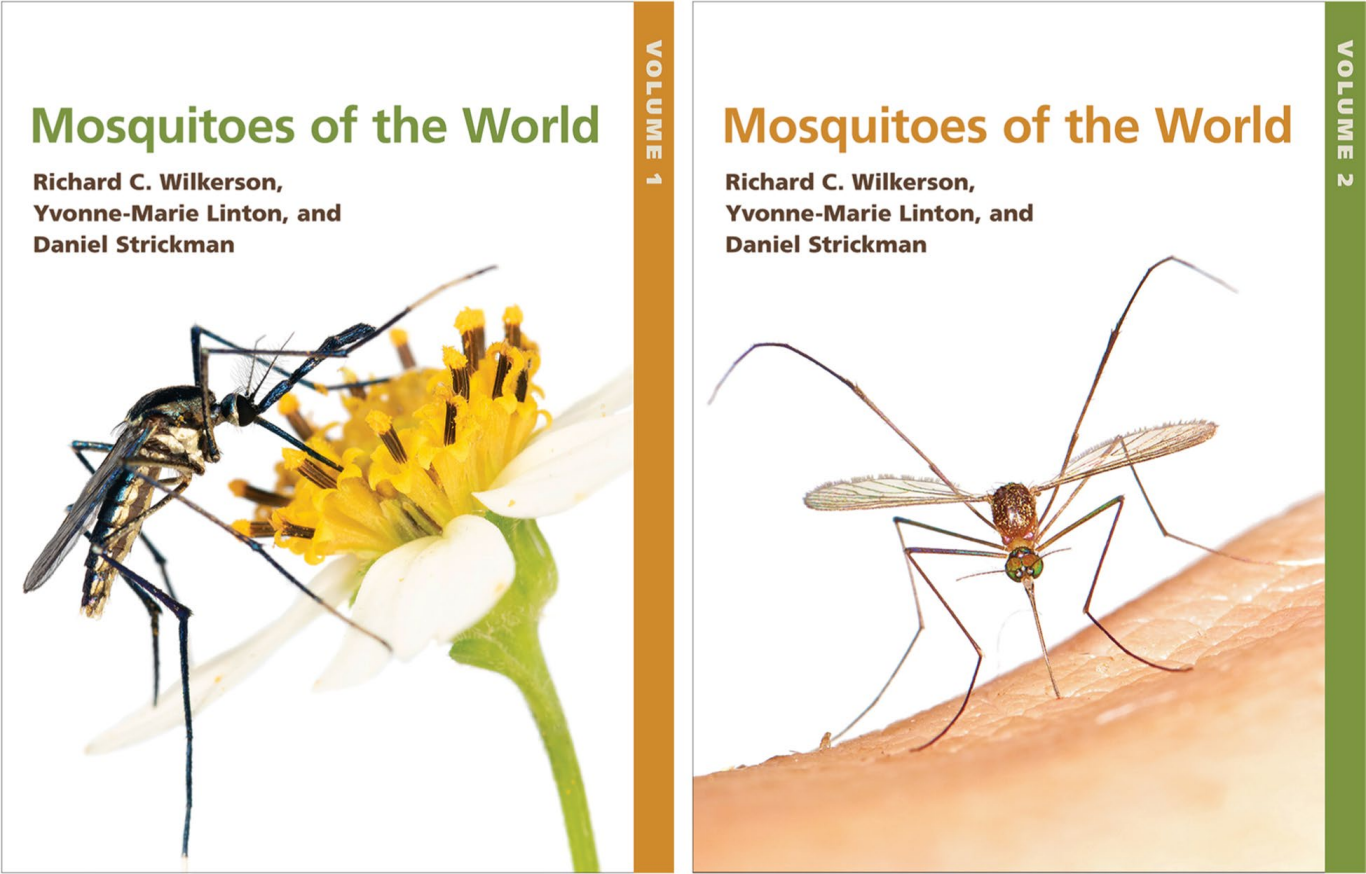
Front cover: “*Mosquitoes of the World*,” Vols. 1 and 2

When a courier rang my doorbell and handed me a big blue bag, I had no idea of what I had just received, except that it was a heavy parcel. Indeed, it was so heavy that, out of curiosity, I decided to weigh it. “*Mosquitoes of the World*,” Vols. 1 and 2 (Fig. 1), weighs an impressive 5.1 kg! Not the kind of scientific literature one carries around with one or puts in one’s backpack during one’s travels. I wondered if I should have accepted the invitation of the editor-in-chief of Parasites and Vectors to undertake this review, as this task would consume a big chunk of my sparsest commodity: time. A sigh of relief must, therefore, have been audible when I started flipping through the pages and it dawned on me that half of this two-volume set consists of a catalog detailing the current taxonomic and systematic status of all validated 3698 species and subspecies, 187 subgenera and 41 genera of mosquitoes. At first sight not the most exciting kind of reading matter, until, that is, I started to take a closer look…

Volume I of “*Mosquitoes of the World*” is divided into two sections. Part I covers mosquito biology in almost 200 pages, and includes ten chapters on a diverse range of topics such as evolution, life cycle, development, feeding, mating, etc. I read a few chapters in full, and concluded that these provide a good starting point for newcomers to the field. Learning about the fundamental mechanisms of biological evolution, from *r*- to* K*-strategists, to reproductive mortality, selection and inheritance, from the perspective of mosquitoes, is interesting, and I learned many new things from this book. For example, I did not know that the last common ancestor of *Drosophila* and mosquitoes (Culicidae) flew on this planet some 260 million years ago. The presented timeline of events of culicid history, which covers almost three entire pages, ends 28 years ago, with the introduction of the Asian tiger mosquito in Italy. In the chapter on nomenclature, the authors point to a critical issue when they state that “The level of knowledge may be decreasing as traditional taxonomy is less and less considered a core part of the biological curriculum, a danger that has been recognized for decades.” This is so true. When trapping mosquitoes out in the field, there’s always that pile of “unidentified” mosquitoes that remains. And only recently have we discovered that some of these “ignored” species may actually play a significant role in disease transmission, even though their abundance may be low [1, 2]. This underscores the importance of nomenclature and supports the authors’ call to train more scientists in the fields of taxonomy and systematics. I liked the list of common names for mosquito genera and the explanations given for assigning certain of them. For example, I did not know that there are genera commonly known as “gang mosquitoes,” “cool mosquitoes,” “royal mosquitoes,” “Maori mosquitoes,” or even “heaven mosquitoes.” Also of interest to me was the seven-page list of recorded invasions of mosquitoes, not surprisingly spearheaded, in terms of frequency, by *Aedes albopictus*, one of the most invasive mosquito species in the world. Noteworthy though, is that the most recent reference cited in this list dates back to 2012. Surely, over the past 9 years, many more invasions—one of which, that of *Aedes japonicus*, happened to have occurred in my own country, the Netherlands in 2013 (8 years ago!) [3]—must have taken place? This list is therefore incomplete. The chapter on mating is interesting, although it does not make reference to some of the more recent studies. The beauty of the variety of mating mechanisms—from swarms to copulation near the blood host, to males of *Deinocerites cancer* that guard female pupae so that they can be the first to mate with the females when they eclose—is fascinating. And although modern genetic control strategies depend to a large extent on the effective mating of released mass-reared specimens, it is surprising how little we still know about this crucial aspect of mosquito life history behavior. The section on the applications of this knowledge is rather limited, and it would have been nice if more of the strategies presently under development had been elaborated on [e.g. clustered regularly interspaced short palindromic repeats (CRISPR)–associated protein 9 (CRISPR/Cas9) is mentioned in only a few sentences, yet this approach is quickly becoming established in this field of research].

Interestingly, the authors decided not to include information on mosquito control. For the sake of completeness, they could have at least included the fundamentals underpinning contemporary control strategies. Moreover, not only is the topic of mosquito control missing, but also monitoring and the vast discipline of mosquito ecology are only marginally addressed. Considering “*Mosquitoes of the World*” as primarily a comprehensive guide to the taxonomy and systematics of mosquitoes is probably the best way to assess the contribution of these volumes to the field. For a more in-depth overview of mosquito biology and control, one should consult Alan Clements’ three-volume “*Biology of Mosquitoes*” [4–6], which together with Mike Service’s classic “*Mosquito Ecology: field sampling methods*” [7], would be a good start for anyone venturing into the world of mosquitoes.

The second part of volume 1 is a marvel for the die-hard mosquito biologist. It comprises a series of beautifully illustrated pages that summarize the diagnostic morphological characters, systematics, distributions, bionomics, associated pathogens and exemplar DNA sequences for each of the 41 genera and 128 globally important mosquito species. When reading about the genus *Anopheles*, with which this section starts, I noticed that the bionomics section reports the recently detected high-altitude wind-borne passive migration of adult mosquitoes of species within this genus. It is somewhat surprising that this recent finding, which is not referenced, was selected from the numerous, influential (review) articles on the bionomics of this genus. The pictures and drawings of mosquito genitals and other morphological features, however, are of a very high standard and quality. It is a joy to flip through the pages and read up on the selected species, for which distribution maps have been included. Not all of this material is easy to understand when you are a novice in mosquito taxonomy, though. For instance, the section on the Asian tiger mosquito’s diagnostic characters includes the following:

“(b) Thorax (side): Antealar area with patch of broad pale scales (AnS); mesepimeron with lower scales (LMSc); paratergite with scales (PaSc), postpronotal scales (PpSc) present; postspiracular scales absent (PoSc); proepisternal scales (PeSc) present; scutal angles (ScA) without pale scales; subspiracular area with broad white scales (SSc).”

This example clearly shows that this material is not for the faint-hearted (and this part only describes the side of the thorax—there is more on the dorsal part of the thorax, the head, the larva, etc.). Although this treatment of the morphological diversity of mosquitoes is accompanied by a glossary of all the morphological terms used, someone new to the field will not find it easy to use.

Volume 2 is the first printed catalog of all described mosquito species (including fossils and synonyms) in over 40 years, and follows Knight and Stone’s seminal work published in 1977 [8]. During the intervening four decades, the number of validated species and subspecies has increased from 3,133 to 3,700: an increase of 16%.

I decided to mine my memory for three mosquito species that crossed my path during my 30 years of mosquito research, and see how “*Mosquitoes of the World*” would help me find details about them. First was *Sabethes belisarioi*, a species that I tried to rear during my PhD because it (supposedly) prefers to land on and bite the nose of humans. It is perhaps also one of the most beautiful mosquitoes in the world, with its magnificent metallic blue color and “feathered” legs (I read that these are called “leg paddles”) that assist in its amazing courtship behavior. Within seconds, Vol. 2 helped me to locate the tribe Sabethini in the contents overview on p. ix, and from the genus *Sabethes* I was directed to p. 993. And there, on that very page, I immediately located *Sabethes belisarioi*. I was then referred back to Vol. 1, p. 278, for a description of the genus, its systematics and distribution, bionomics, associated pathogens and diagnostic characters. Although the two volumes comprise 1332 pages, I reached my “destination” within seconds, which reveals the ease with which one can maneuver through the catalog. I discovered that species of the genus *Sabethes* are called “canopy mosquitoes,” and that *Sabethes belisarioi* also has an informal name: the “Belisario Brazilian canopy mosquito.” The type location for this species is Bicudos, Minas Gerais, Brazil, although the location where the type specimen was deposited is unknown. Seven references accompany the description, one of which, “Mattingly 1971a,” surprised me. When I looked this up in the reference list, it referred to “Contributions to the mosquito fauna of South East Asia.” To my knowledge, there are no *Sabethes belisarioi* in Southeast Asia, thus I presume that this reference was cited in error.

The second species I dug up from memory was *Anopheles sundaicus*, a species that featured in an extensive review of the taxonomy and bionomics of Indonesian anophelines that I worked on during my MSc. [9]. On looking it up, I was sent from the index (p. 680) back to Vol. 1 (p. 376), again within seconds. The common name of this species complex is “Sunda Indonesian nail mosquito,” which apparently now consists of five distinct forms. It has long played a dominant role in malaria transmission in Indonesia, and is one of the species that, amongst the many types of anophelines in that country, was targeted through a process called “species sanitation,” which focused on the management of the habitat of the prime malaria vectors and not that of all anopheline species. Obviously, the ability to recognize the different species then became imperative. “*Mosquitoes of the World*” mentions the crepuscular biting activity and lists some of the preferred breeding sites of *An. sundaicus*, but its extensive breeding in coastal areas with fish ponds and irrigated rice fields is not mentioned.

Lastly, I selected the Asian tiger mosquito, *Aedes albopictus*, and again within seconds I found myself reading about the type location, Calcutta (West Bengal), India, and that the type specimen is kept in the United Sates National Museum in Washington DC. There was also half a page listing the countries from which it has been reported, followed by half a page of references. I work with this species every day in the Maldives, where we are trying to eliminate its populations from small coral islands. The descriptions of its bionomics in terms of breeding sites, biting habits, diapausing and dessication-resistant eggs are all highly informative and accurate. Some references to guide the reader further would have been useful, though.

As the most complete reference work on mosquitoes ever produced, “*Mosquitoes of the World*” is an extremely useful resource for entomologists, public health professionals and epidemiologists, and for inclusion in reference libraries. The fully updated catalog, which includes many new species, puts this work at the forefront of mosquito taxonomy and systematics. The style, layout and organization fit the book’s purpose, as evidenced by my searches for the three mosquito species mentioned above.

The authors are world experts in this field, and it is sad to note that one of them, Dr. Dan Strickman, did not live to see this massive work in print. “*Mosquitoes of the World*” will surely be considered one of the many great achievements of his career [10]. According to the authors, their rationale for writing “*Mosquitoes of the World*” was “to facilitate the future fight against mosquitoes through a holistic understanding of their diversity, systematics, and associated biology.” There is no doubt that they succeeded in doing just that, and I foresee that this work will serve as the gold standard for decades to come in the quest to “know thy enemy” before going into battle against mosquitoes. Mosquito taxonomy remains a grossly underrated and underrepresented discipline that few develop a passion for, but virtually all in the field depend on when trying to understand the bug that they are dealing with. “*Mosquitoes of the World*” will hopefully enthuse a new generation of biologists to venture into this fascinating field!

## Data Availability

Not applicable.
